# Genotypic Distribution of Hepatitis C Virus in Thailand and Southeast Asia

**DOI:** 10.1371/journal.pone.0126764

**Published:** 2015-05-11

**Authors:** Rujipat Wasitthankasem, Sompong Vongpunsawad, Nipaporn Siripon, Chutima Suya, Phrutsada Chulothok, Kasemporn Chaiear, Pairaya Rujirojindakul, Sawan Kanjana, Apiradee Theamboonlers, Pisit Tangkijvanich, Yong Poovorawan

**Affiliations:** 1 Center of Excellence in Clinical Virology, Faculty of Medicine, Chulalongkorn University, Bangkok, Thailand; 2 Chiangrai Prachanukroh Hospital, Chiang Rai, Thailand; 3 Udon Thani Hospital, Udon Thani, Thailand; 4 Department of Pathology, Faculty of Medicine, Prince of Songkla University, Songkhla, Thailand; 5 Regional Blood Center XI Nakhorn Si Thammarat, Thai Red Cross Society, Thung Song District, Nakhon Si Thammarat, Thailand; 6 Department of Biochemistry, Faculty of Medicine, Chulalongkorn University, Bangkok, Thailand; Kobe University, JAPAN

## Abstract

The majority of hepatitis C virus (HCV) infection results in chronic infection, which can lead to liver cirrhosis and hepatocellular carcinoma. Global burden of hepatitis C virus (HCV) is estimated at 150 million individuals, or 3% of the world’s population. The distribution of the seven major genotypes of HCV varies with geographical regions. Since Asia has a high incidence of HCV, we assessed the distribution of HCV genotypes in Thailand and Southeast Asia. From 588 HCV-positive samples obtained throughout Thailand, we characterized the HCV 5’ untranslated region, Core, and NS5B regions by nested PCR. Nucleotide sequences obtained from both the Core and NS5B of these isolates were subjected to phylogenetic analysis, and genotypes were assigned using published reference genotypes. Results were compared to the epidemiological data of HCV genotypes identified within Southeast Asian. Among the HCV subtypes characterized in the Thai samples, subtype 3a was the most predominant (36.4%), followed by 1a (19.9%), 1b (12.6%), 3b (9.7%) and 2a (0.5%). While genotype 1 was prevalent throughout Thailand (27–36%), genotype 3 was more common in the south. Genotype 6 (20.9%) constituted subtype 6f (7.8%), 6n (7.7%), 6i (3.4%), 6j and 6m (0.7% each), 6c (0.3%), 6v and 6xa (0.2% each) and its prevalence was significantly lower in southern Thailand compared to the north and northeast (p = 0.027 and *p* = 0.030, respectively). Within Southeast Asia, high prevalence of genotype 6 occurred in northern countries such as Myanmar, Laos, and Vietnam, while genotype 3 was prevalent in Thailand and Malaysia. Island nations of Singapore, Indonesia and Philippines demonstrated prevalence of genotype 1. This study further provides regional HCV genotype information that may be useful in fostering sound public health policy and tracking future patterns of HCV spread.

## Introduction

Hepatitis C virus (HCV) infection is a global public health problem with approximately 130 to 150 million infected individuals worldwide [1]. Most HCV infection will lead to chronic hepatitis, cirrhosis and hepatocellular carcinoma, which result in 500000 deaths annually from HCV-related liver diseases. Prevalence of HCV varies depending on the country and region. HCV seroprevalence is < 2% in developed countries, but ≤ 15% in developing countries [2]. Unsafe medical procedures prior to HCV awareness, blood transfusion, and unsterile needle-sharing among intravenous drug users (IVDU) are major modes of HCV transmission and has contributed to the rapid spread of some common strains [2,3]. As a result, the distribution of HCV genotypes and subtypes differ substantially. For instance, genotypes 1, 2, and 3 are widely distributed while other genotypes are confined to certain geographical area. Genotype 4 prevails in Africa and Middle East, but genotypes 5 and 6 are endemic in South Africa and Southeast Asia, respectively. A newly identified genotype 7 has been isolated from a Congolese immigrant in Canada [4,5].

Knowledge about HCV genotypes is not only important for appropriate treatment regimen, but their epidemiological data can reveal transmission activity and migration movement of infected individuals from the endemic area. Among patients with genotype 1 or 4, the treatment response rate to conventional antiviral therapy of pegylated-interferon and ribavirin is lower than with genotypes 2 and 3 [6]. The treatment for genotypes 1 and 4 also requires longer duration of drug administration. Regional spread of some HCV genotypes is associated with particular transmission factors. Subtype 1b spread effectively via blood transfusion, while subtype 1a and 3a became predominant through injecting drug used [3,7]. Subtypes 4a and 1b are common in Egypt and Japan, respectively, due to the onset of iatrogenic injection of anti-schistosomiasis campaign during the 20^th^ century [8,9]. Migration from an endemic area to new regions is also thought to be responsible for changing the HCV genotype landscape. An example is the emergence of genotype 6 in industrialized countries such as Canada and Australia, which is genetically similar to the most isolated genotype of Southeast Asian linage [5,10].

Even within Southeast Asia, common genotypes and prevalence varies geographically. Genotype 6 is dominant in South China, Myanmar, Laos, Vietnam and Cambodia [11–15] while Genotype 3 is common in Thailand and Malaysia [16,17]. Surprisingly, genotype 1 became the major genotype in Singapore, Indonesia and Philippines [18–20], possibly due to its introduction from western countries during or after World War II [7].

Past epidemiological studies of HCV in Thailand provided inconsistent data due to the selection of the population and areas under study. A seroprevalence study of randomly selected individuals from four geographically distinct provinces showed approximately 2.2% of the individuals had anti-HCV, with subtype 3a (51.1%), subtype 1b (26.7%), genotype 6 (8.9%), subtype 1a (6.7%), and subtype 3b (2.2%) being most common [16]. First-time blood donors screened by the National Blood Center in Bangkok showed a lower HCV seroprevalence of 0.98–0.51% [21,22]. Not surprisingly, high-risk group such as IVDU demonstrated 70–90% seroprevalence [21,23]. When specific regions of the country were examined, blood donors from central Thailand showed high frequency of subtype 3a (up to 70%) and low frequency of genotype 6 (2.6%) [21], while donors from the north showed lower frequency of 3a (33.3%) and higher frequency of 6 (31%) [24]. In addition, there is insufficient epidemiological data from southern Thailand. Screening for anti-HCV antibody from large sample size generally result in only a few hundred RNA-positive samples available for genome characterization [16,23,25]. Furthermore, there is a lack of integrated national and regional database of HCV prevalence. Therefore, this study aims to evaluate regional burden of HCV within Thailand and among Southeast Asian countries.

## Materials and Methods

### Sample collection

**Fig 1:**
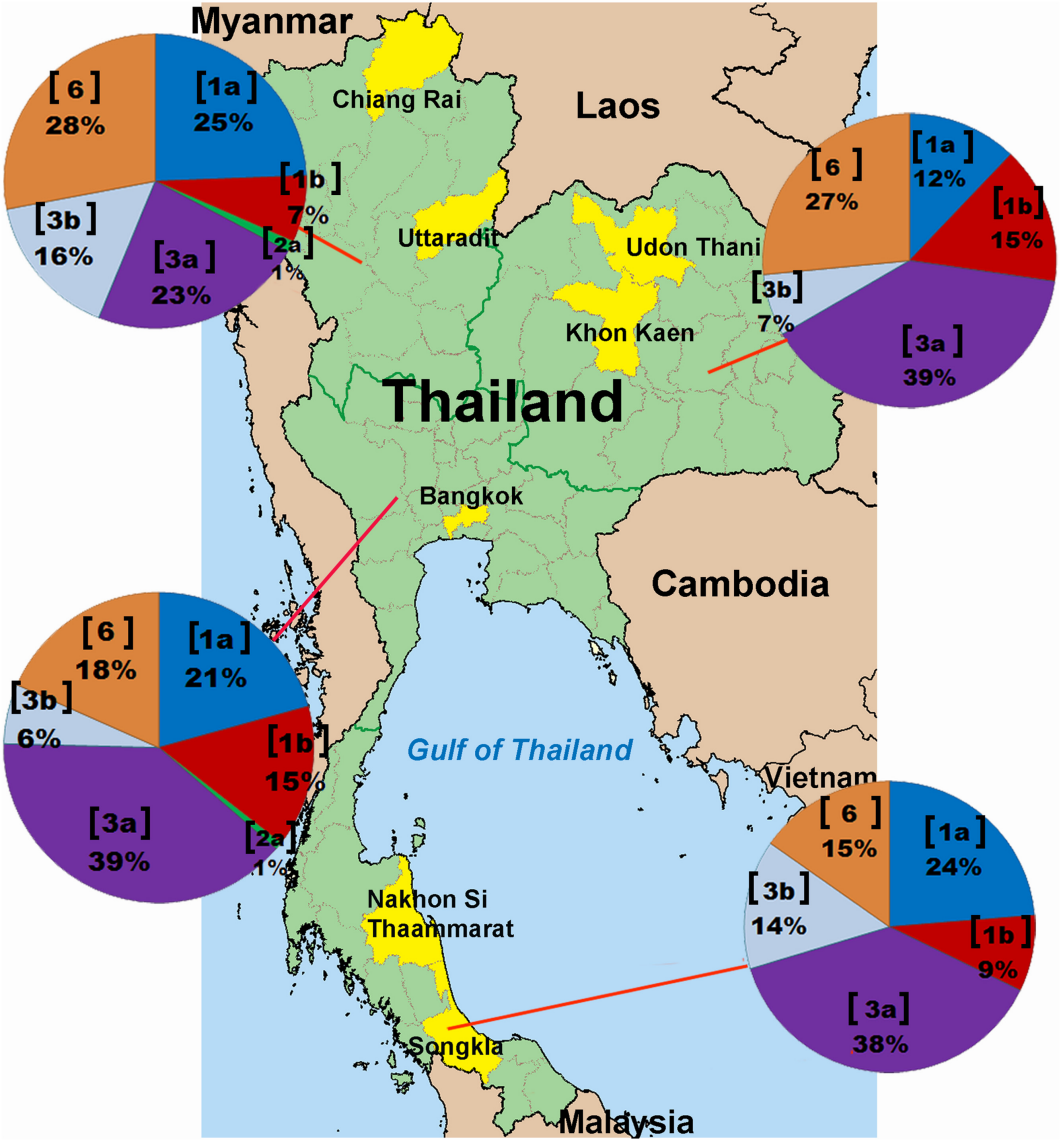
Distribution of HCV genotypes and subtypes in the 4 regions of Thailand. Pie charts indicate the genotypic distribution in the North, Northeast, Central and South based on the analysis of 588 samples. The genotype or subtype is indicated in a bracket, accompanied by the proportion in percentage.

In total, 588 blood samples were collected from individuals who attended outpatient clinic or donated blood from 2007 to 2012. In all, 132 individuals from the Northeast (Udon Thani Hospital in Udon Thani province and Chum Phae Hospital in Khon Kaen province), 118 from the South (Songklanagarind University Hospital in Songkhla province and Maharaj Nakhon Si Thammarat Hospital in Nakhon Si Thammarat province), 82 from the North (Chiang Rai Prachanukroh Hospital in Chiang Rai province and Uttaradit Hospital in Uttaradit province) and 256 from the Central region (Chulalongkorn Memorial Hospital in Bangkok) were included (Fig 1). Information gathered such as age, gender, and collection date were kept confidential and all samples were number-coded and anonymous. The study protocol was approved by the Institutional Review Board (IRB 307/54) of the Faculty of Medicine, Chulalongkorn University and conducted in compliance with the principles of the Declaration of Helsinki. Inform consents were waived because all samples were treated as anonymous.

### Viral extraction and PCR amplification

Viral HCV RNA was extracted from anti-HCV positive serum samples by guanidine thiocyanate method [26]. cDNAs were generated from viral RNA using ImProm-II Reverse Transcriptase (Promega, Madison, WI) according to the manufacturer’s instructions. HCV infection was performed by detecting 5’UTR region using RT-PCR with 2X Perfect Tag PlusMasterMix (5 PRIME, Gaithersburg, MD). For viral genotype assignment, samples positive for HCV 5’UTR were subsequently analyzed for the Core and NS5B regions. Nested PCRs were employed as followed. The 5’UTR outer primers were OC1 and OC2 and the inner primers were and IC4 (S1 Table). The Core region outer primers were 954F and 410R and the inner primers were 953F and 951R (S1 Table) [27]. First and second amplification reactions for both 5’UTR and Core were as followed: pre-incubation at 94°C for 3 min., 40 cycles of denaturation at 94°C for 1 min., annealing at 49°C for 1 min., extension at 72°C for 1.30 min. and a final extension step at 72°C for 7 min.

Degenerate primer sets for NS5B region consisted of outer primers NS5B_F1 and NS5B_R1 and inner primers NS5B_F2 and NS5BR2 (S1 Table). PCR conditions for NS5B were identical to those for 5’UTR and Core, except nested PCR annealing temperature was changed to 52°C.

### Sequencing and Phylogenetic analysis

The PCR products of partial Core and NS5B were purified (ExpinGel SV, GeneAll Biotechnology, Seoul, Korea) and subjected to direct sequencing (First BASE Laboratories, Selangor, Malaysia). Sequences were analyzed with Chromas LITE (v2.01), BioEdit v.5.0.9 (Ibis Therapeutics, Carlsbad, CA), and SeqManPro(DNASTAR, Madison, WI), and subjected to BLASTN search (http://www.ncbi.nlm.nih.gov).

Genotypes of HCV isolates were assigned based on the phylogenetic analysis of both Core and NS5B sequences. Partial Core or NS5B sequences were aligned with reference sequences retrieved from GenBank Database using ClustalX v.2.1 [28]. Sequence lengths used for the alignment ranged between 275 to 304 nucleotides for the Core gene and 292 to360 nucleotides for the NS5B gene. The Core and NS5B phylogenetic trees were generated from the dataset for each region (North, Central, Northeast and South). Neighbor-joining trees of Core and NS5B alignments were estimated by using Kimura’s two parameter method implemented in MEGA v.6.0 [29]. Reliability of the phylogenetic trees was assessed by 1000 bootstrap resampling. Reference sequences [subtype] used in this study were: [1a] M62321, M67463; [1b] D90208, M58335; [1c] D14853; [2a] AB047639, D00944; [2b] D10988, AB030907; [2c] D50409; [3a] D17763, D28917; [3b] D49374; [4a] Y11604; [5a] Y13184, AF064490; [6a] Y12083, AY859526; [6b] D84262; [6c] EF424629; [6d] D84263; [6e] DQ314805; [6f] DQ835760; [6g] D63822; [6h] D84265; [6i] DQ835770; [6j] DQ835769; [6k] D84264; [6l] EF424628; [6m] DQ835767; [6n] DQ278894, DQ835768, FU246939; [6o] EF424627; [6p] EF424626; [6q] EF424625; [6r] EU408328; [6s] EU408329 [6t] EF632071, FU246939; [6u] EU246940; [6v] EU158186, EU798760; [6w] DQ278892, EU643834; and [6xa] EU408330, EU408332. All sequences isolated in this study were submitted to GenBank database and the accession numbers were KP323417-KP324281. The rest of nucleotide sequences were reported previously [30,31].

### Data analysis

Distribution of HCV genotype was calculated in proportion to each region of Thailand. Genotypic distributions of HCV in Southeast Asian countries were extrapolated from previous reports [11–13,16–20,32–50]. The Chi-square test was used to compare categorical variables. Post Hoc ANOVA with Bonferroni correction was used to compare differences between group means. P-value less than 0.05 was considered statistically significant. All statistical analyses were calculated by using SPSS for Window version 11.5 (SPSS, Chicago, IL).

## Results

### Distribution of HCV genotypes in Thailand

Among the 588 samples representing 4 different geographical areas of Thailand, approximately 40% (n = 256) were from the Central region (Table 1, S1–S4 Figs). Since most individuals were male (gender ratio 2.7:1), they contributed to a significantly higher prevalence of HCV infection than female in all regions (*p* < 0.000). The overall age range was 12 to 73 years (mean 41.5 ± 10.6 years). The South represented the youngest mean age, which was statistically significant compared to those in the Central (*p* < 0.000) and the Northeast (*p* = 0.040) regions. No statistically significant differences in the mean age were observed among genotypes 1, 3, and 6 (*p* = 0.493, S2 Table). Subtype 6m (54.0 ± 4.0 years) and subtype 2a (34.7 ± 5.8 years) showed the oldest and youngest age, respectively (S3 Table). Differences in the mean age were significant among subtypes (*p* = 0.0005). We found that the difference in age was significant between 6f (46.8 ± 8.3 year) and 1a (40.3 ± 11.1 year; *p* = 0.027), 6f and 3b (38.6 ± 10.9 years; *p* = 0.007), and 6f and 6n (37.7 ± 9.1 years; *p* = 0.003).

**Table 1:** Characteristics and genotypes of HCV found in Thailand.

	North	Northeast	Central	South	Total
**Sample**	82	132	256	118	588
**Mean age (SD)**	40.0 (10.4)	41.5 (9.4)	43.6 (11.4)	37.8 (9.0)	41.5 (10.6)
**Sex (M/F)**	70/12	98/34	163/93	96/22	427/161
**Genotype (%)**	**26 (31.7)**	**36 (27.3)**	**91 (35.5)**	**38 (32.2)**	**191 (32.5)**
1a	20 (24.4)	16 (12.1)	53 (20.7)	28 (23.7)	117 (19.9)
1b	6 (7.3)	20 (15.2)	38 (14.8)	10 (8.5)	74 (12.6)
**Genotype 2 (%)**	**1 (1.2)**	**0 (0.0)**	**2 (0.8)**	**0 (0)**	**3 (0.5)**
2a	1 (1.2)	0 (0.0)	2 (0.8)	0 (0)	3 (0.5)
**Genotype 3 (%)**	**32 (39.0)**	**61 (46.2)**	**116 (45.3)**	**62 (52.5)**	**271 (46.1)**
3a	19 (23.2)	52 (39.4)	98 (38.3)	45 (38.1)	214 (36.4)
3b	13 (15.9)	9 (6.8)	18 (7.0)	17 (14.4)	57 (9.7)
**Genotype 6 (%)**	**23 (28.0)**	**35 (26.5)**	**47 (18.4)**	**18 (15.3)**	**123 (20.9)**
6c	2 (2.4)	0 (0.0)	0 (0.0)	0 (0.0)	2 (0.3)
6f	7 (8.5)	15 (11.3)	21 (8.2)	3 (2.5)	46 (7.8)
6i	1 (1.2)	12 (9.1)	7 (2.7)	0 (0.0)	20 (3.4)
6j	0 (0.0)	0 (0.0)	4 (1.6)	0 (0.0)	4 (0.7)
6m	4 (4.9)	0 (0.0)	0 (0.0)	0 (0.0)	4 (0.7)
6n	8 (9.8)	7 (5.3)	15 (5.9)	15 (12.7)	45 (7.7)
6v	0 (0.0)	1 (0.8)	0 (0.0)	0 (0.0)	1 (0.2)
6xa	1 (1.2)	0 (0.0)	0 (0.0)	0 (0.0)	1 (0.2)

Although we identified 4 HCV genotypes (1, 2, 3 and 6) and 13 subtypes (1a, 1b, 3a, 3b, 2a, 6c, 6f, 6i, 6j, 6m, 6n, 6v and 6xa) in the Thai samples (S1–S4 Figs), their distribution varied depending on the regions of Thailand (Table 1). In decreasing order, the most common HCV strains were genotype 3 (46.1%), genotype 1 (32.5%), genotype 6 (20.9%) and genotype 2 (0.5%). Subtype 3a (36.4%) comprised the most predominant subtype, followed by subtype 3b (9.7%). The overall frequency of genotype 1 was 27.3% to 35.5%, including subtype 1a (19.9%) and subtype 1b (12.6%). Genotype 6 showed very high variations, which included eight subtypes: 6c (0.3%), 6f (7.8%), 6i (3.4%), 6j (0.7), 6m (0.7%), 6n (7.7%), 6v (0.2%) and 6xa (0.2%). Lastly, subtype 2a (0.5%) was least frequently observed.

Genotype 3, particularly subtypes 3a and 3b, was the most predominant HCV in all regions of Thailand with the lowest frequency in the North (39%) and highest in the South (52.2%) (Fig 1). Distribution of genotype 6 was also significantly different among the 4 geographical regions (*p* = 0.04), with the frequency and diversity appeared to decrease from North to South. We found that the proportion of genotype 6 identified in the South was significantly lower than that in the North (28%) and the Northeast (26.5%) (*p* = 0.027 and *p* = 0.030, respectively). Although at least 8 subtypes (6c, 6f, 6i, 6j, 6m, 6n, 6v and 6xa) circulated throughout the country, only subtypes 6c and 6m were found in the North (*p* = 0.006 and *p* < 0.000, respectively). Two novel subtypes (6v and 6xa) were also identified in the Northeast and the North, respectively. Lastly, we detected only a few samples containing subtype 2a (0.5%).

### Distribution of HCV genotypes in Southeast Asia (SEA)

**Fig 2:**
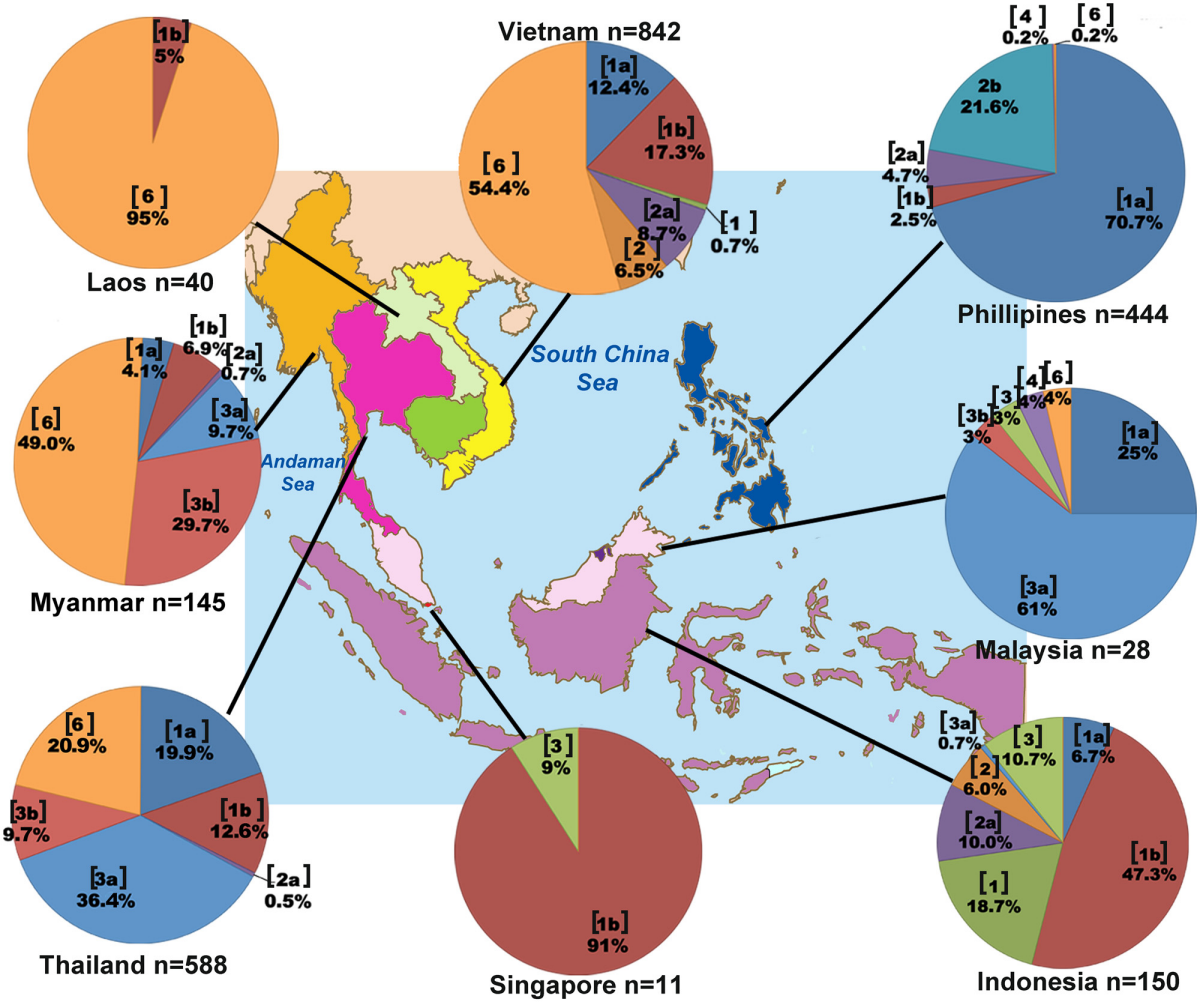
Distribution of HCV genotypes in Southeast Asian region compiled from published literatures. Pie charts indicate the genotypic distribution found in each country. The genotype or subtype is indicated in a bracket, accompanied by the proportion in percentage [11–13, 17–20].

To compare the distribution of HCV genotypes found in Thailand to those in Southeast Asia region, we compiled data from several reports on the overall genotypic prevalence in Thailand and eight neighboring countries, which included Myanmar, Laos, Vietnam, Cambodia, Malaysia, Singapore, Indonesia and the Philippines (Table 2, Fig 2). Insufficient data on Brunei precluded it from the analysis.

**Table 2:** Distribution of HCV genotypes in Southeast Asian countries.

Country	Year	Sample No.	Sample group	Genotyping method	Genotype number (%)						Reference
					1	2	3	4	6	UN	
**Myanmar**	2001	24	Liver disease	Primer specific PCR	4(16.7)	0	18(75.0)	0	0	2(8.3)	Nakai et al. 2001 [32]
	2004	110	Blood donor	Phylogentic	35 (31.8)	0	52(47.3)	0	23(20.9)	0	Shinji et al. 2004 [33]
	2007	145	Normal population	Phylogentic	16(11.0)	1(0.7)	57(39.3)	0	71(49.0)	0	Lwin et al. 2007 [11]
	2011	15	Migrant worker	Phylogentic	2(13.4)	0	9(60)	0	4(26.6)	0	Akkarathamrongsin et al. 2011 [34]
	2014	4	US-bound refugee	Phylogentic	0	0	1(25)	0	3(75)	0	Mixson-Hayden et al. 2014 [35]
**Laos**	2009	16	Liver disease	Phylogentic	0	0	0	0	16(100)	0	Pybus et al. 2009 [36]
	2011	40	Blood donor	Phylogentic	2(5.0)	0	0	0	38(95.0)	0	Hubchen et al. 2011 [12]
**Vietnam**	2009	70	Blood donor	Phylogentic	33(47.1)	0	4(5.7)	0	33(47.1)	0	Pham et al. 2009 [37]
	2010	114	IVDU	Phylogentic	75(65.8)	1(0.9)	10(8.8)	0	28(24.5)	0	Tanimoto et al. 2010 [38]
	2011	842	Blood donor	Nucleotide BLAST	256(30.4)	128(15.2)	0	0	458(54.4)	0	Pham et al. 2011 [13]
	2012	277	High risk groups^a^	Phylogentic	166(59.9)	1(0.4)	5(1.8)	0	105(37.9)	0	Dunford et al. 2012 [39]
	2014	9	Normal population	Not mentioned	1(11.1)	1(11.1)	1(11.1)	0	6 (66.7)	0	Do et al. 2014 [40]
	2014	236	Blood donor and Liver disease	Phylogentic	77(32.6)	34(14.4)	0	0	125(53.0)	0	Li et al. 2014 [41]
**Cambodia**	2011	25	Migrant worker	Phylogentic	6(24.0)	0	5(20.0)	0	14(56.0)	0	Akkarathamroongsin et al. 2011 [34]
	2014	11	Normal population	Not mentioned	3(27.3)	0	0	0	6(54.5)	2(18.2)	Yamada et al. 2014 [14]
**Thailand**	2007	45	Blood donor	Phylogentic	16(35.6)	1(2.2)	24(53.3)	0	4(8.9)	0	Sunanchaikarn et al. 2007 [16]
	2014	588	Blood donor and Liver disease	Phylogentic	191(32.5)	3(0.5)	271(46.1)	0	123(20.9)	0	This study
**Malaysia**	2012	28	Hemodialysis patient	Phylogentic	7(25.0)	0	19(67.9)	1(3.6)	1(3.6)	0	Hairul et al. 2012 [17]
	2013	37	Liver disease	Nucleotide BLAST	10(27.0)	0	27(73)	0	0	0	Mohamed et al. 2013 [42]
	2014	17	Liver disease	Nucleotide BLAST	5(29.4)	0	12(70.6)	0	0	0	Mohamed et al. 2014 [43]
**Singapore**	1995	16	Liver disease	Nucleotide homology	11(68.8)	2(12.5)	2(12.5)	1(6.2)	0	0	Ng et al. 1995 [44]
	1995	11	Not mentioned	Aminoacid similarity	10(90.9)	0	1(9.1)	0	0	0	Greene et al. 1995 [18]
**Indonesia**	2000	57	Blood donor	Primer specific PCR	39(60.9)	12(18.8)	8(12.5)	0	0	5(7.8)	Inoue et al. 2000 [45]
	2008	104	Blood donor and liver disease	Phylogentic	64(61.5)	21(20.2)	18(17.3)	1(1.0)	0	0	Utama et al. 2008 [46]
	2010	150	Liver disease	Phylogentic	109(72.7)	24(16.0)	17(11.3)	0	0	0	Utama et al 2010 [19]
	2012	44	HIV patient	Phylogentic	28(63.6)	0	12(27.3)	3(6.8)	1(2.3)	0	Anggorowati et al. 2012 [47]
	2013	30	Prisoner	Phylogentic	20(66.7)	0	8(26.6)	2(6.7)	0	0	Prasetyo et al. 2013 [48]
	2014	99	HIV patient	Nucleotide sequence homology	57(57.6)	2(2.0)	39(39.4)	1(1.0)	0	0	Juniastuti et al. 2014 [49]
**Philippines**	2005	23	IVDU	Phylogentic	15(65.2)	8(34.8)	0	0	0	0	Agdamag et al. 2005 [50]
	2009	444	IVDU and dialysis patient	Phylogentic	325(73.2)	117(26.4)	0	1(0.2)	1(0.2)	0	Kageyama et al. 2009 [20]

The number of samples examined for each study with assignable HCV genotype was included.

^a^Intravenous drug user, commercial sex worker, dialysis worker and multi-transfused patient.

Fig 2 represented HCV genotypic distribution in each SEA deduced from studies with largest sample size and contained HCV genotype classification by direct sequencing or phylogenetic analyses in each respective country (S4 Table). Five HCV genotypes (1, 2, 3, 4 and 6) were observed in the region (Fig 2, Table 2). Genotype 1 frequencies were high in Singapore (90.9%, n = 11), Indonesia (72.7%, n = 150) and the Philippines (73.2%, n = 444), while genotype 2 was less common [18–20]. Vietnam appeared to have the most diverse genotype 2 subtypes (2a, 2c, 2i, 2j and 2k) [13]. Genotype 3 was most frequently found in Thailand and Malaysia. Available data appeared to suggest that it is the predominant genotype in Malaysia (67.9 to 73.0%, Table 2) [17,42,43]. Genotype 4 subtype 4a was reported in Malaysia, Singapore, Indonesia, and the Philippines (Table 2) [17,20,44,46–49]. Genotype 6 was most commonly found in Myanmar, Laos and Vietnam, and presented the most diverse subtypes (≥ 18) in Southeast Asia. Twelve subtypes (6a, 6c, 6e, 6f, 6h, 6k, 6l, 6n, 6o, 6p, 6r and 6t) were reported in Vietnam alone [11–13]. Although genotype 6 was most common in Laos (95.8%, n = 40), the majority of isolates have not been assigned subtypes [12].

## Discussion

Due to substantial genetic diversity intrinsic to RNA viruses, HCV has been classified into 7 genotypes with 67 confirmed and 20 provisional subtypes [4]. Differences in the geographic distribution of HCV genotypes also underscore its complex evolutionary past. Genotypes 1 and 3 (especially subtypes 1a, 1b and 3a) are the first and second most prevalent strains worldwide, respectively [10]. Other genotypes are found in smaller proportion and relatively restricted to certain geographical areas. In this study, we examined HCV genotypes in four regions of Thailand. Subtype 3a was the predominant subtype nationally (36.4%) and regionally (ranging from 23.2% to 39.4%) in agreement with previous findings [16,25]. The distribution of genotype 6 demonstrated high prevalence in the North and Northeast in comparison to the South (*p* = 0.027 and *p* = 0.030, respectively). In addition, we detected a number of genotype 6 subtypes including the novel subtype 6xa, which was formerly assigned to 6u subtype [4]. We noted that only one sample was discordant for the subtype but otherwise concordant for genotype (1b for Core and 1a for NS5B). Finally, frequency of subtype 2a was low, consistent with a previous study [16].

Distribution patterns of HCV genotype in northern Thailand correlated with a hospital-based study in Chiang Mai province that found genotype 3 was dominant, followed by genotypes 1 and 6 [51]. Various new subtypes (6c, 6f, 6i, 6j, 6m, 6n, 6v and additional unassigned subtypes) were also identified in HCV-infected individuals from several locations in the North (S5 Table) [24]. A prior study done in the Northeast demonstrated substantially higher frequency of subtype 3a (76.5%) than that found in our cohort (39.4%), possibly due to provincial differences since that study did not identify any subtype 3b (S5 Table) [52]. The higher rate of HCV infection was generally observed among IVDU (70%) compared to methamphetamine and inhalant drug users (12.0%-21.1%) in which most were infected with subtype 3a (73.1%) linked to IVDU transmission [21,53,54]. In addition, evolutionary analysis suggests that subtype 3a found its way into industrialized countries via IVDU [3]. Although IVDU may have initially been responsible for the introduction of HCV into Thailand, viral spread was subsequently exacerbated by iatrogenic medical procedures and contamined blood supply [31]. Patterns of HCV genotypes observed in the Central and the South were quite similar (subtype 3a, 1a, 1b or 3b and 6 variants), although the geographic distribution of genotype 6 differed among the 4 regions (*p* = 0.040) and showed significantly lower frequency in the South than the North (*p* = 0.027). We note that the distribution of HCV genotypes in southern Thailand was similar to Malaysia in which genotype 3 was most prevalent followed by genotypes 1 and 6 [17].

At least 9 subtypes of HCV genotype 6 (6a, 6d, 6e, 6h, 6k, 6l, 6o, 6p and 6t) were reported in Vietnam (S4 Table) [13]. In Laos, 7 subtypes are known to exist (6b, 6h, 6i, 6j, 6l, 6o and 6q), while many more remained unclassified [12,36]. In Myanmar, at least 4 subtypes (6f, 6m, 6n and 6xa) have been reported [30,33,55], while studies in Cambodia have identified at least 6 subtypes (6e, 6f, 6f, 6p, 6q, 6r, 6s) [5,14,34]. Moreover, novel subtypes (6v and 6xa) were first isolated as unassigned subtypes from this region [55,56]. Genotype 6 is relatively uncommon in Malaysia where only one whole genome of subtype 6n had been isolated in 2013 from an IVDU individual with HIV/HCV co-infection [57]. Epidemiological evidence suggests that genotype 6 has been prevalent in southern China and Hong Kong due to imported cases, which then effectively spread among IVDU and the general population via blood transfusion [15,58–60]. The high prevalence and genetic diversity therefore support the argument that genotype 6 may have long circulated or even originated in Southeast Asia [36].

The available data on the prevalence of HCV genotypes among Southeast Asian countries suggest three general trends (Table 2). First, there is a preponderance of genotype 6 in the lower mainland China and upper Southeast Asian countries, including Myanmar, Laos, Vietnam, Cambodia, and northern Thailand [11–14,59]. This genotype contributed roughly 20% to 50% in Myanmar depending on the studies [11,33]. The frequency was greater than 50% in Vietnam and limited data showed highest rate in Laos [12,13]. One recent study also found HCV genotype 6 dominant (54.5%) in the Cambodian population [14], comparable to the rate of 56% found among Cambodian workers in Thailand [34].

Second, HCV genotype 3 appears to dominate the central plain of Thailand and the Malay Peninsula (Table 2) [16,17]. Subtype 3a was frequent in the IVDU group; it has been suggested that this subtype spread primarily via needle-sharing during the Vietnam War. It eventually entered the general population and became endemic in Thailand through modern medicine and blood transfusion [3,21,31]. Coincidently, genotype 3 is also common in the Indian subcontinent including India, Pakistan and Nepal [61–63]. Whole genome sequencing of several genotype 3 subtypes estimated that genotype 3 may have originated 780 years ago in Africa and entered South Asia around 450 years ago by the Arabian slave traders [64]. Since then, this genotype has circulated in India and diverged into several subtypes, including 3a which originated 99 years ago and disseminated to the United Kingdom and other European countries [65]. Moreover, subtype 3a identified in Thailand is genetically similar to Indian isolates, perhaps as a result of the long history of trade and migration between Thailand and the Indian subcontinent [31]. Further characterization of the whole genome of subtype 3a found in Thailand may confirm this hypothesis.

The third trend is the predominance of genotype 1 in Singapore and further south including the islands of Indonesia and the Philippines (Table 2) [18–20]. Specifically, subtype 1a was common in the Philippines while 1b was common in Singapore and Indonesia. The former genotype has been associated with IVDU transmission, while the latter through blood transfusion [3,53]. Genotype 1 is hypothesized to have originated in Africa [66]. Phylogenetic analysis revealed ancestral sub-genotype 1 in West and Central Africa, whereas subtypes 1a and 1b isolated in industrial countries had diverged from the African linage approximately 135 to 112 years ago [67]. This period coincided with the trans-Atlantic slave trade from Africa to North America and Europe. Subtypes 1a and 1b further disseminated after World War II through blood transfusion, iatrogenic procedure, and injection of drug stimulant or IVDU [7,10,68]. Based on NS5B sequences of subtypes 1a and 1b isolated in different parts of the world, phylogenetic analyses suggest that these subtypes had first diverged in developed countries and were subsequently introduced into developing countries [7]. Furthermore, phylogenetic tree in that study showed that subtype 1a isolated in the Philippines appeared to be directly related to the U.S. strains.

There are several limitations in our study. The de-identified samples were conveniently obtained from out-patient clinics and from the blood bank through blood donation. Clinical information therefore were not available to infer factors associated with the observed frequency and types of HCV found in this study. Individuals from which the samples were obtained may not be representative of the general population in Thailand. Due to the diversity in the lifestyle, behavioral risk, healthcare, diet, and religion among residents of Southeast Asian countries, the prevalence of different HCV genotypes and subtypes found may not be generalizable to other parts of the world. Some countries in Southeast Asia lacked published studies on HCV. If studies were available, the population sampled may have been small, and as a result some genotypes and subtypes may be underestimated or not represented. Nonetheless, our data provided valuable insight into the present burden of HCV infections in Thailand relative to other Southeast Asian countries.

Despite the high diversity of HCV in Thailand and Southeast Asia, patterns of genotypic distribution emerged. The impending economic integration of the Association of Southeast Asian Nations in 2015, which would allow non-restricted travel among residents of the member states and unprecedented free trade of goods and services similar to the establishment of the European Union, may alter the future landscape of viral diseases in this region. Therefore, this study may provide justifications for sound public health policy, including the surveillance of transmission pattern in the future.

## Supporting Information

S1 Table Nucleotide sequences of primers used in this study. Nucleotide position numbering of each primer was based on the reference strain H77 (GenBank accession number M62321).
(DOCX)

S2 Table Age distribution of different HCV genotypes. (DOCX)

S3 Table The mean age for each HCV subtype identified in samples collected in Thailand. Comparisons among the subtypes showed significant differences with 6f and are indicated by the *p*-values.
(DOCX)

S4 Table Distribution of HCV genotypes and subtypes among 8 Southeast Asian countries. This data was presented in the pie chart of Fig 2.
(DOCX)

S5 Table Comparison of published HCV studies and this study on the prevalence of HCV genotypes and subtypes in Thailand. (DOCX)

S1 Fig Phylogenetic tree based on the Core (left) and NS5B (right) sequences of samples collected from northern Thailand. Black circles indicate reference genotypes with accession numbers and genotypes.
(TIF)

S2 Fig Phylogenetic tree based on Core (left) and NS5B (right) sequences of samples collected from central Thailand. Black circles indicate reference genotypes with accession numbers and genotypes.
(TIF)

S3 Fig Phylogenetic tree based on Core (left) and NS5B (right) sequences of samples collected from northeastern Thailand. Black circles indicate reference genotypes with accession numbers and genotypes.
(TIF)

S4 Fig Phylogenetic tree based on Core (left) and NS5B (right) sequences of samples collected from southern Thailand. Black circles indicate reference genotypes with accession numbers and genotypes.
(TIF)
