# Supplementary material for: Genotypic Distribution of Hepatitis C Virus in Thailand and Southeast Asia
Source: PLoS One. 2015 May 11;10(5):e0126764. doi: 10.1371/journal.pone.0126764 (PMC4427325; doi:10.1371/journal.pone.0126764)
Supplement: S1 Table — Nucleotide position numbering of each primer was based on the reference strain H77 (GenBank accession number M62321). (DOCX) [file pone.0126764.s001.docx]

**Supporting Information**

**S1 Table. Nucleotide sequences of primers used in this study**.

| **HCV region** | **Primer Name** | **Direction** | **Position** | **Sequence** |
| --- | --- | --- | --- | --- |
| 5’UTR | OC1 | Forward | 18- 37 | 5’-GCCGACACTCCACCATGAAT-3 |
|  | OC2 | Reverse | 328-347 | 5’-CATGGTGCACGGTCTACGAG-3’ |
|  | IC3 | Forward | 51-71 | 5’-GGAACTACTGTCTTCACGCAG-3’ |
|  | IC4 | Reverse | 290-310 | 5’-TCGCAAGCACCCTATCAGGCA-3’ |
| Core^a^ | 954F | Forward | 288-311 | 5’-ACTGCCTGATAGGGTGCTTGCGAG-3’ |
|  | 410R | Reverse | 732-751 | 5’-ATGTACCCCATGAGGTCGGC-3’ |
|  | 953F | Forward | 321-344 | 5’-AGGTCTCGTAGACCGTGCATCATG-3’ |
|  | 951R | Reverse | 705-725 | 5’-CACTGTRAGGGTATCGATGAC-3’ |
| NS5B | NS5BF1 | Forward | 7999-8020 | 5’-CAATWSMMACBACCATCATGGC-3’ |
|  | NS5BR1 | Reverse | 8805-8825 | 5’-CCAGGARTTRACTGGAGTGTG-3’ |
|  | NS5BF2 | Forward | 8159-8181 | 5’-GATGGGHHSBKCMTAYGGATTCC-3’ |
|  | NSB5R2 | Reverse | 8611-8630 | 5’-CATAGCNTCCGTGAANGCTC-3’ |

Nucleotide position numbering of each primer was based on the reference strain H77 (GenBank accession number M62321).

^a^As previously described by Mellor et al. 1995 [27].
