# Supplementary material for: Genotypic Distribution of Hepatitis C Virus in Thailand and Southeast Asia
Source: PLoS One. 2015 May 11;10(5):e0126764. doi: 10.1371/journal.pone.0126764 (PMC4427325; doi:10.1371/journal.pone.0126764)
Supplement: S2 Table — (DOCX) [file pone.0126764.s002.docx]

**S2 Table**. **Age distribution of different HCV genotypes**.

|  | **Number of samples** | **Mean age(SD)** | **Minimum age** | **Maximum age** |
| --- | --- | --- | --- | --- |
| **Genotype 1** | 187 | 41.4(10.7) | 17 | 69 |
| **Genotype 2** | 3 | 34.7(5.8) | 28 | 38 |
| **Genotype 3** | 265 | 41.3(10.8) | 12 | 73 |
| **Genotype 6** | 121 | 42.5(1.0) | 17 | 65 |
| **Total** | 576^a^ | 41.5(10.6) | 12 | 73 |

^a^Twelve samples lacked data on age.
