# Supplementary material for: Genotypic Distribution of Hepatitis C Virus in Thailand and Southeast Asia
Source: PLoS One. 2015 May 11;10(5):e0126764. doi: 10.1371/journal.pone.0126764 (PMC4427325; doi:10.1371/journal.pone.0126764)
Supplement: S3 Table — Comparisons among the subtypes showed significant differences with 6f and are indicated by the p-values. (DOCX) [file pone.0126764.s003.docx]

**S3 Table**. **The mean age for each HCV subtype identified in samples collected in Thailand.**

| **HCV subtype** | **Sample number** | **Mean age (SD)** | **Minimum age** | **Maximum age** | ***p*-value** |
| --- | --- | --- | --- | --- | --- |
| 1a | 114 | 40.3(11.1) | 18 | 69 | 0.027 |
| 1b | 73 | 43.0 (9.9) | 17 | 66 |  |
| 2a | 3 | 34.7(5.8) | 28 | 38 |  |
| 3a | 212 | 41.9(10.7) | 12 | 73 |  |
| 3b | 53 | 38.6(10.9) | 17 | 60 | 0.007 |
| 6c | 2 | 46.0(14.1) | 36 | 56 |  |
| 6f | 45 | 46.8(8.3) | 31 | 65 |  |
| 6i | 20 | 39.9(10.4) | 18 | 54 |  |
| 6j | 4 | 44.0(10.9) | 28 | 52 |  |
| 6m | 4 | 54.0(4.0) | 48 | 56 |  |
| 6n | 44 | 37.7(9.1) | 17 | 60 | 0.003 |
| 6u | 1 | 36.0(0.0) | 36 | 36 |  |
| 6v | 1 | 58.0(0.0) | 58 | 58 |  |
| Total | 576^a^ | 41.5(10.6) | 12 | 73 |  |

Comparisons among the subtypes showed significant differences with 6f and are indicated by the p-values.

^a^Twelve samples had no data on age.
