# Supplementary material for: Genotypic Distribution of Hepatitis C Virus in Thailand and Southeast Asia
Source: PLoS One. 2015 May 11;10(5):e0126764. doi: 10.1371/journal.pone.0126764 (PMC4427325; doi:10.1371/journal.pone.0126764)
Supplement: S4 Table — This data was presented in the pie chart of Fig 2. (DOCX) [file pone.0126764.s004.docx]

**S4 Table.** **Distribution of HCV genotypes and subtypes among 8 Southeast Asian countries.**

|  | **Myanmar** | **Laos** | **Vietnam** | **Thailand** | **Malaysia** | **Singapore** | **Indonesia** | **Philippines** |
| --- | --- | --- | --- | --- | --- | --- | --- | --- |
| **Genotype 1** | **11.0** | **4.2** | **30.4** | **32.5** | **25.0** | **90.9** | **72.7** | **73.2** |
| **1a** | 4.1 | 0.0 | 12.4 | 19.9 | 25.0 | 0.0 | 6.7 | 70.7 |
| **1b** | 6.9 | 4.2 | 17.3 | 12.6 | 0.0 | 90.9 | 47.3 | 2.5 |
| **1c** | 0.0 | 0.0 | 0.00 | 0.00 | 0.0 | 0.0 | 18.7 | 0.0 |
| **1e** | 0.0 | 0.0 | 0.7 | 0.00 | 0.0 | 0.0 | 0.0 | 0.0 |
| **Genotype 2** | **0.7** | **0.0** | **15.2** | **0.5** | **0.0** | **0.0** | **16.0** | **26.4** |
| **2a** | 0.7 | 0.0 | 8.7 | 0.5 | 0.0 | 0.0 | 10.0 | 4.8 |
| **2b** | 0.0 | 0.0 | 0.0 | 0.0 | 0.0 | 0.0 | 0.0 | 21.6 |
| **2c** | 0.0 | 0.0 | 4.3 | 0.0 | 0.0 | 0.0 | 0.0 | 0.0 |
| **2e** | 0.0 | 0.0 | 0.0 | 0.0 | 0.0 | 0.0 | 5.3 | 0.0 |
| **2f** | 0.0 | 0.0 | 0.0 | 0.0 | 0.0 | 0.0 | 0.7 | 0.0 |
| **2i** | 0.0 | 0.0 | 1.8 | 0.0 | 0.0 | 0.0 | 0.0 | 0.0 |
| **2j** | 0.0 | 0.0 | 0.2 | 0.0 | 0.0 | 0.0 | 0.0 | 0.0 |
| **2k** | 0.0 | 0.0 | 0.2 | 0.0 | 0.0 | 0.0 | 0.0 | 0.0 |
| **Genotype 3** | **39.3** | **0.0** | **0.0** | **46.1** | **67.8** | **9.1** | **11.3** | **0.0** |
| **3a** | 9.7 | 0.0 | 0.0 | 36.4 | 60.7 | 0.0 | 0.7 | 0.0 |
| **3b** | 29.6 | 0.0 | 0.0 | 9.7 | 3.5 | 0.0 | 0.0 | 0.0 |
| **3k** | 0.0 | 0.0 | 0.0 | 0.0 | 0.0 | 9.1 | 10.6 | 0.0 |
| **3** | 0.0 | 0.0 | 0.0 | 0.0 | 3.6 | 0.0 | 0.0 | 0.0 |
| **Genotype 4** | **0.0** | **0.0** | **0.0** | **0.0** | **3.6** | **0.0** | **0.0** | **0.2** |
| **4a** | 0.0 | 0.0 | 0.0 | 0.0 | 3.6 | 0.0 | 0.0 | 0.2 |
| **Genotype 6** | **49.0** | **95.8** | **54.4** | **20.9** | **3.6** | **0.0** | **0.0** | **0.2** |
| **6a** | 0.0 | 0.0 | 23.6 | 0.0 | 0.0 | 0.0 | 0.0 | 0.0 |
| **6b** | 0.0 | 6.2 | 0.0 | 0.0 | 0.0 | 0.0 | 0.0 | 0.0 |
| **6c** | 0.0 | 0.0 | 0.4 | 0.3 | 0.0 | 0.0 | 0.0 | 0.0 |
| **6e** | 0.0 | 0.0 | 22.0 | 0.0 | 0.0 | 0.0 | 0.0 | 0.0 |
| **6f** | 0.0 | 0.0 | 0.1 | 7.8 | 0.0 | 0.0 | 0.0 | 0.0 |
| **6h** | 0.0 | 2.1 | 1.2 | 0.0 | 0.0 | 0.0 | 0.0 | 0.0 |
| **6i** | 0.0 | 0.0 | 0.0 | 3.4 | 0.0 | 0.0 | 0.0 | 0.0 |
| **6j** | 0.0 | 0.0 | 0.0 | 0.7 | 0.0 | 0.0 | 0.0 | 0.0 |
| **6k** | 0.0 | 2.1 | 0.5 | 0.0 | 0.0 | 0.0 | 0.0 | 0.0 |
| **6l** | 0.0 | 2.1 | 3.2 | 0.0 | 0.0 | 0.0 | 0.0 | 0.0 |
| **6m** | 9.0 | 0.0 | 0.0 | 0.7 | 0.0 | 0.0 | 0.0 | 0.0 |
| **6n** | 38.6 | 2.1 | 0.1 | 7.6 | 0.0 | 0.0 | 0.0 | 0.0 |
| **6o** | 0.0 | 0.0 | 1.5 | 0.0 | 0.0 | 0.0 | 0.0 | 0.0 |
| **6p** | 0.0 | 2.1 | 1.0 | 0.0 | 0.0 | 0.0 | 0.0 | 0.0 |
| **6r** | 0.0 | 0.0 | 0.1 | 0.0 | 0.0 | 0.0 | 0.0 | 0.0 |
| **6t** | 0.0 | 0.0 | 0.7 | 0.0 | 0.0 | 0.0 | 0.0 | 0.0 |
| **6v** | 0.0 | 0.0 | 0.0 | 0.2 | 0.0 | 0.0 | 0.0 | 0.0 |
| **6xa** | 0.0 | 0.0 | 0.0 | 0.2 | 0.0 | 0.0 | 0.0 | 0.0 |
| **6.00** | 1.4 | 79.1 | 0.0 | 0.0 | 3.6 | 0.0 | 0.0 | 0.2 |
| **Total** | 100.0 | 100.0 | 100.0 | 100.0 | 100.0 | 100.0 | 100.0 | 100.00 |
| **Reference** | 11 | 12 | 13 | This study | 17 | 18 | 19 | 20 |

This data was presented in the pie chart of Fig. 2.
