# Supplementary material for: Genotypic Distribution of Hepatitis C Virus in Thailand and Southeast Asia
Source: PLoS One. 2015 May 11;10(5):e0126764. doi: 10.1371/journal.pone.0126764 (PMC4427325; doi:10.1371/journal.pone.0126764)
Supplement: S5 Table — (DOCX) [file pone.0126764.s005.docx]

**S5 Table.** **Comparison of published HCV studies and this study on the prevalence of HCV genotypes and subtypes in Thailand**.

| **Reference** | **Theamboonlers et al. 2002(26)** | **Verachaiet al. 2002(21)** | | **Sunanchaikarn et al. 2007(16)** | **Jutavijittum et al. 2009(24)** | **Kumthip et al. 2014(51)** | **Barusrux et al., 2014(52)** | **This study** | | | | |
| --- | --- | --- | --- | --- | --- | --- | --- | --- | --- | --- | --- | --- |
| **Sampling location** | Central | Central | | 4 regions | North | North | Northeast | North | Northeast | Central | South | 4 regions |
| **Sampling group** | Blood donor | Blood donor | IVDU | Blood donor | Blood donor | Liver disease | Blood donor | Blood donor and liver disease | | | | |
| **Sample number** | 77 | 152 | 94 | 45 | 122 | 158 | 98 | 82 | 132 | 256 | 118 | 588 |
| **Genotype 1 (%)** | **39.0** | **11.7** | **24.3** | **35.6** | **28.7** | **31.0** | **15.3** | **31.7** | **27.3** | **35.5** | **32.2** | **32.5** |
| **1a** | 20.8 | 0 | 5.3 | 6.7 | 14.8 | 12.6 | 8.2 | 24.4 | 12.1 | 20.7 | 23.7 | 19.9 |
| **1b** | 18.2 | 11.7 | 19 | 28.9 | 13.9 | 18.4 | 7.1 | 7.3 | 15.2 | 14.8 | 8.5 | 12.6 |
| **Genotype 2 (%)** | **0** | **0** | **0** | **2.2** | **0** | **0** | **0** | **1.2** | **0.0** | **0.8** | **0.0** | **0.5** |
| **2a** | 0 | 0 | 0 | 2.2 | 0 | 0 | 0 | 1.2 | 0.0 | 0.8 | 0.0 | 0.5 |
| **Genotype 3 (%)** | **44.1** | **75.5** | **73.1** | **53.3** | **40.2** | **54.5** | **76.5** | **39.1** | **46.2** | **45.3** | **52.5** | **46.1** |
| **3a** | 37.6 | 75.5 | 72.4 | 48.9 | 33.6 | 45 | 76.5 | 23.2 | 39.4 | 38.3 | 38.1 | 36.4 |
| **3b** | 6.5 | 0 | 0.7 | 4.4 | **6.6** | 9.5 | 0 | 15.9 | 6.8 | 7.0 | 14.4 | 9.7 |
| **Genotype 6 (%)** | **16.9** | **12.8** | **2.6** | **8.8** | **31.0** | **14.5** | **8.1** | **28.0** | **26.5** | **18.4** | **15.2** | **21.0** |
| **6a** | 0 | 8.5 | 2.6 | 0 | 0 | 0.6 | 0 | 0 | 0 | 0 | 0 | 0 |
| **6c** | 0 | 0 | 0 | 0 | 0.8 | 0 | 0 | 2.4 | 0 | 0 | 0 | 0.3 |
| **6f** | 1.3 | 0 | 0 | 4.4 | 8.2 | 12.6 | 2.0 | 8.5 | 11.3 | 8.2 | 2.5 | 7.8 |
| **6i** | 3.9 | 0 | 0 | 0 | 0.8 | 0.6 | 4.1 | 1.2 | 9.1 | 2.7 | 0 | 3.4 |
| **6j** | 0 | 0 | 0 | 0 | 1.6 | 0 | 0 | 0 | 0 | 1.6 | 0 | 0.7 |
| **6m** | 0 | 0 | 0 | 0 | 4.9 | 0 | 0 | 4.9 | 0 | 0 | 0 | 0.7 |
| **6n** | 11.7 | 0 | 0 | 4.4 | 12.3 | 0.7 | 2 | 9.8 | 5.3 | 5.9 | 12.7 | 7.7 |
| **6v** | 0 | 0 | 0 | 0 | 1.6 | 0 | 0 | 0 | 0.8 | 0 | 0 | 0.2 |
| **6xa** | 0 | 0 | 0 | 0 | 0 | 0 | 0 | 1.2 | 0 | 0 | 0 | 0.2 |
| **6 variants (%)** | 0 | 0 | 0 | 0 | 0.8 | 0 | 0 | 0 | 0 | 0 | 0 | 0 |
| **Unclassified (%)** | 0 | 4.3 | 0 | 0 | 0 | 0 | 0 | 0 | 0 | 0 | 0 | 0 |
