# Supplementary figures and images for: Genotypic Distribution of Hepatitis C Virus in Thailand and Southeast Asia
Source: PLoS One. 2015 May 11;10(5):e0126764. doi: 10.1371/journal.pone.0126764 (PMC4427325; doi:10.1371/journal.pone.0126764)

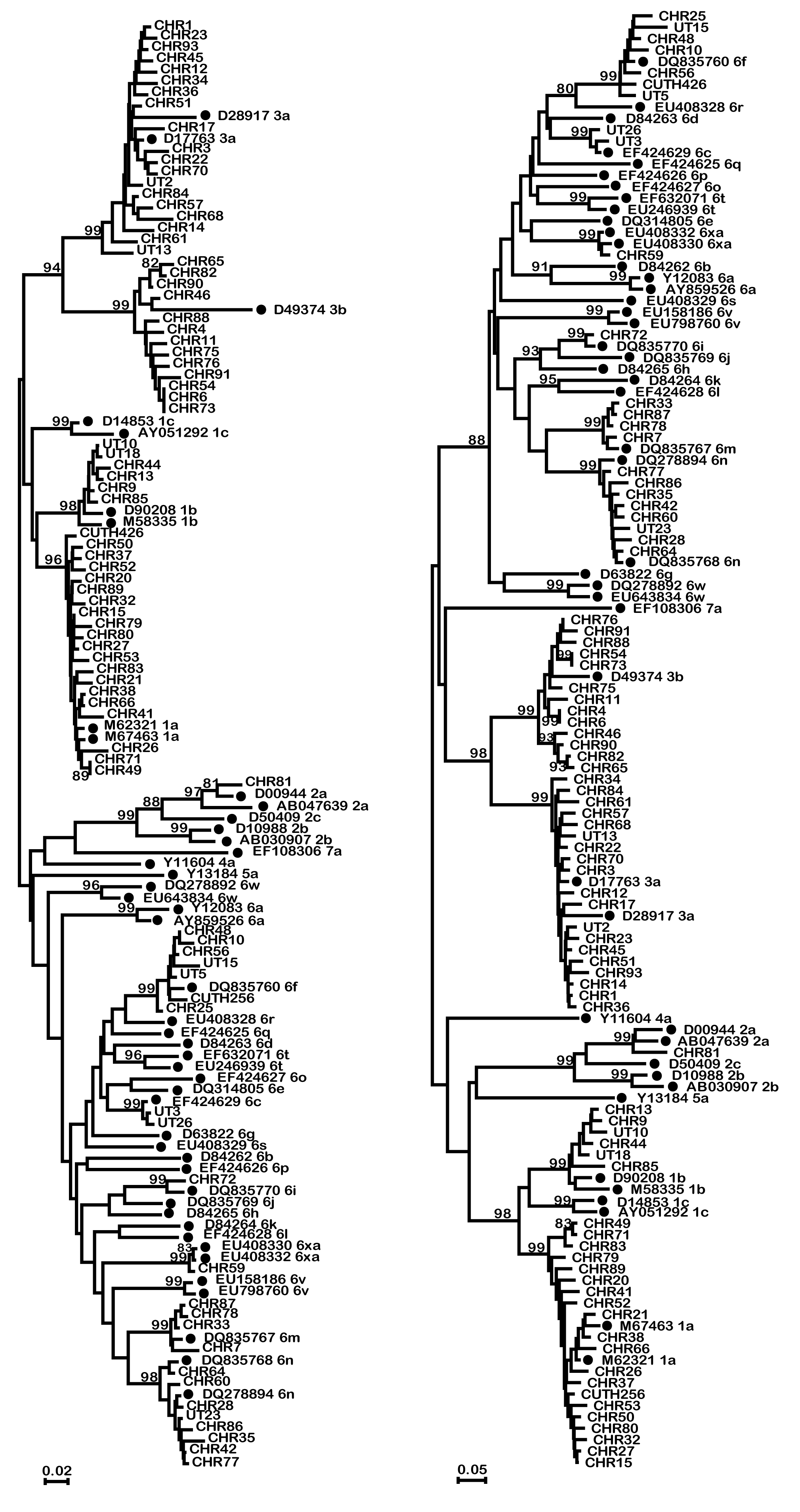

Supplement: S1 Fig — Black circles indicate reference genotypes with accession numbers and genotypes. (TIF) [file pone.0126764.s006.tif]

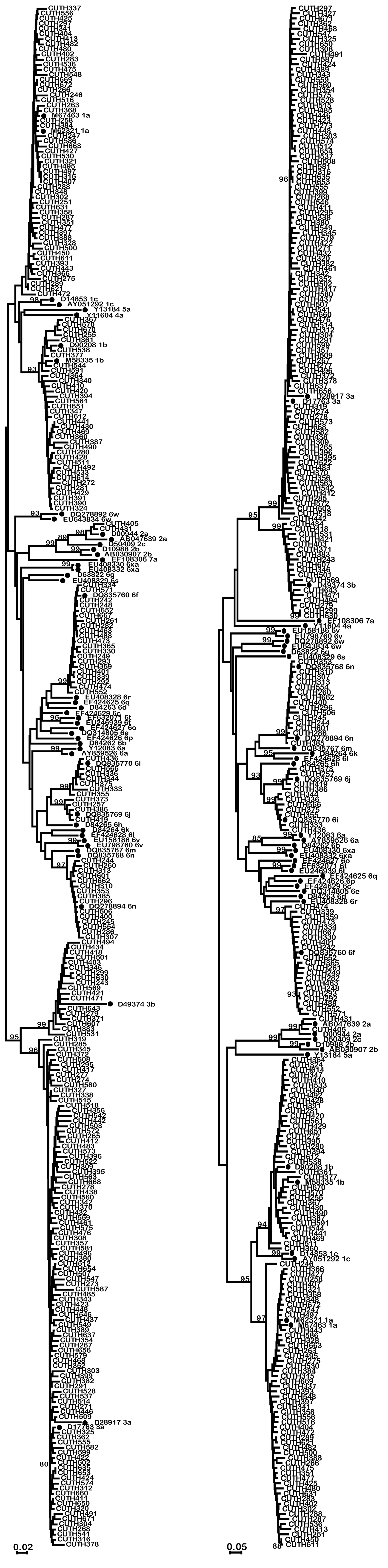

Supplement: S2 Fig — Black circles indicate reference genotypes with accession numbers and genotypes. (TIF) [file pone.0126764.s007.tif]

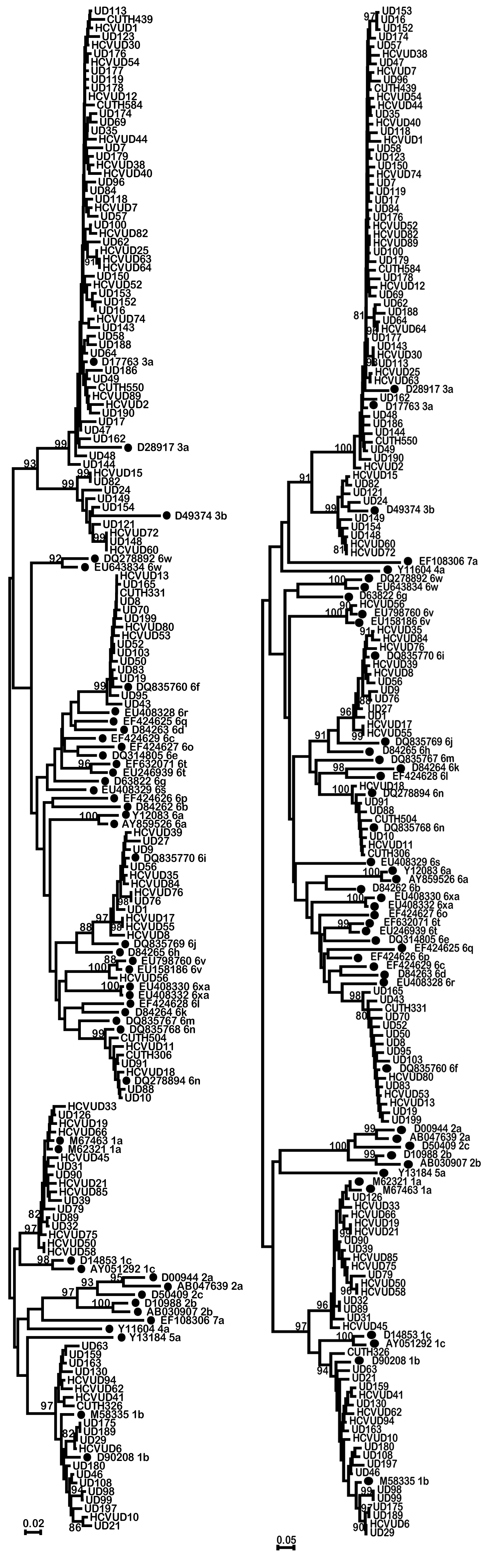

Supplement: S3 Fig — Black circles indicate reference genotypes with accession numbers and genotypes. (TIF) [file pone.0126764.s008.tif]

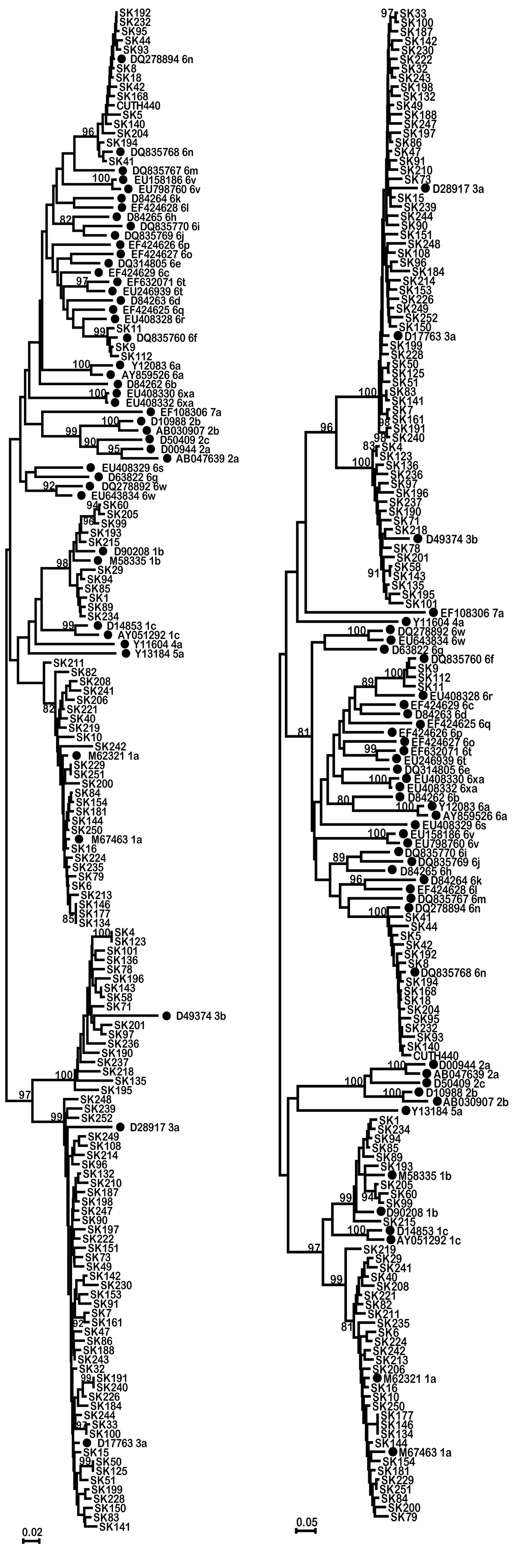

Supplement: S4 Fig — Black circles indicate reference genotypes with accession numbers and genotypes. (TIF) [file pone.0126764.s009.tif]
